# Public health round-up

**DOI:** 10.2471/BLT.20.010320

**Published:** 2020-03-01

**International response to new coronavirus outbreak**

Hong Kong street cleaners receive a briefing on COVID-19 symptoms and prevention from a *Médecins sans Frontières* educator as part of the international response to the outbreak of the new coronavirus which began in Wuhan, China in December.

**Figure:**
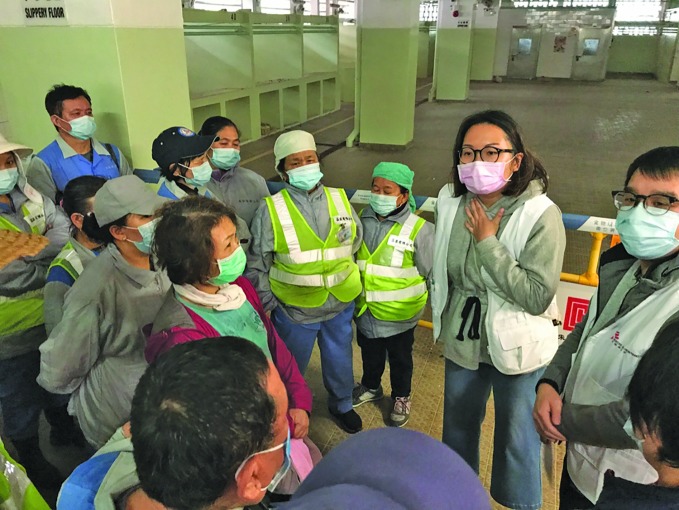
MSF/Shuk Lim Cheung

## Novel coronavirus response

A plan to prevent further spread of the new coronavirus (COVID-19) virus and to protect states with weaker health systems was launched by the international community on 5 February.

The Strategic Preparedness and Response Plan (SPRP) sets out activities and resources needed by international health organizations globally, including the World Health Organization (WHO), to implement priority public health measures in support of countries to prepare and respond to COVID-19 for the period February-April 2020.

The objectives of the plan are to limit human-to-human transmission of the virus, identify, isolate and care for patients early, communicate critical risk and event information, minimize social and economic impact, reduce virus spread from animal sources, and accelerate priority research and innovation.

In early January WHO activated a team to coordinate and facilitate information-sharing on research elements needed for the response. The team will implement the WHO R&D Blueprint, a strategy for accelerating research and development activities during epidemics. The R&D Blueprint includes scenarios for “pathogen x” – a previously unknown pathogen – such as COVID-19. WHO convened a meeting of experts in Geneva on 11 and 12 February to set research and development priorities for coronavirus treatment, diagnostics, vaccines, infection prevention and community measures to combat the outbreak.

The total estimated resources required to implement the SPRP over a three months period is USD 675.5 million.

The outbreak was first declared in Wuhan, China in December 2019. As of 11 February, 43 101 people had been confirmed to be infected with the virus of whom 1018 had died. To date, most reported cases (42 708) and deaths (1017) are in China.

Following the advice of the Emergency Committee, WHO Director-General declared the outbreak of novel coronavirus (COVID-19) a Public Health Emergency of International Concern on 30 January.

http://bit.ly/2SxBMyr

## Syrian hostilities intensify

Fighting in northwest Syria has intensified since the beginning of the year, giving rise to a massive movement of people within the country. As of 11 February, a total of 689 000 people had been displaced.

The fighting has also led to the closure of more than 70 health facilities, leaving the population with limited access to health care. So far in 2020, three separate attacks on health care services have been verified, all in the northwest, causing 10 deaths.

“To fill the gap created by closed health facilities, we are revising our referral network, trying to sustain stocks of medicines for those with non-communicable diseases and supporting the relocation of some of the health facilities,” said Rick Brennan, WHO’s regional emergency director.

WHO is also increasing the number of mobile clinics that can follow the movements of the displaced.

A fragile immunization network put in place by WHO and partners has also been disrupted, increasing the population’s exposure to infectious diseases.

A 2-month supply of essential medicine has been prepositioned on either side of Syria’s border with Turkey, but there is concern that demand will be far greater than supply.

“What is striking about this escalation is that the enormous humanitarian needs are being largely ignored by the international media and governments,” Brennan said. He called for a renewed international commitment to ending the crisis.

http://bit.ly/2SeqSib

## Collaboration on essential diagnostics

WHO and the Foundation for Innovative New Diagnostics (FIND) have established a formal collaboration on universal access to essential diagnostics. Announced on 10 February, the aim of the collaboration is to close diagnostic gaps at country level and bolster disease surveillance that will inform public health initiatives and enhance outbreak preparedness and response.

Accurate and timely diagnosis is critical to achieving universal health coverage, but can be a weak link in the delivery of health services. This is especially true in primary healthcare settings in low- and middle-income countries.

http://bit.ly/3bqCFS5

## Cancer incidence set to rise

If current trends continue, the world will see a 60% increase in cancer cases over the next two decades. The greatest increase (an estimated 81%) in new cases will occur in low- and middle-income countries, where survival rates are currently lowest.

These sobering predictions are presented in the *WHO report on cancer: setting priorities, investing wisely and providing care for all*, which was published on 4 February along with the International Agency for Research on Cancer’s *World cancer report: cancer research for cancer prevention*.

According to the WHO report the increase is largely due to lack of health services to prevent, diagnose and treat cancers. In 2019, more than 90% of high-income countries reported that comprehensive treatment services for cancer were available in the public health system, compared to less than 15% of low-income countries. The report highlights a range of proven interventions to prevent new cancer cases.

http://bit.ly/2SiEJCV

## Female genital mutilation’s cost

WHO launched a female genital mutilation (FGM) cost calculator, which combines data on health risk associated with FGM, health costs and national FGM prevalence to analyze the economic impact on national health services of treating the consequences of FGM. The FGM Cost Calculator was launched on 6 February, this year’s International Day of Zero Tolerance for FGM.

The data, available for 27 of the 30 countries where FGM is practiced, will strengthen the economic argument of community activists, policy makers, programme planners and donors working to end this human rights violation.

FGM includes procedures that intentionally alter or cause injury to the female genital organs for non-medical reasons. An extreme form of gender discrimination, it is recognized internationally as a violation of the human rights of girls and women, including the right to health, security and physical integrity, and the right to be free from torture and cruel, inhuman or degrading treatment.

Rates of FGM are declining, but because of population growth, the number of girls and women mutilated is forecast to rise significantly in the next 15 years. An estimated 200 million girls and women are living with the effects of genital mutilation, most of whom live in 30 countries in the African, Eastern Mediterranean and South-East Asian Regions.

http://bit.ly/2SllEzW

## WHO appoints Regional Directors

The WHO Executive Board re-appointed Dr Matshidiso Moeti for a second term as WHO Regional Director for Africa and Dr Hans Kluge as Regional Director for Europe.

Dr Moeti was first elected as WHO Regional Director for Africa on 1 February 2015 and is the first woman to hold the position.

Dr Kluge has over 25 years of experience in public health and was previously Director of the Division of Health Systems and Public Health at WHO/Europe.

The Executive Board met at WHO headquarters in Geneva between 3-8 February 2020 to agree on the agenda and resolutions to be considered at this year’s World Health Assembly.

http://bit.ly/2Sgh7yK

**Cover photo**

Instructor Joseph K. Mwangi watches as student Stanley Simwa practices delivering a baby in the Kenya Medical Training College Skills Lab, Nairobi. 2020 is the International Year of the Nurse and Midwife. Nine million more nurses and midwives are needed to make progress towards universal health coverage.

**Figure:**
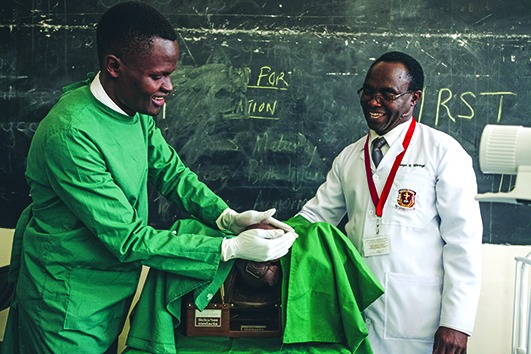
WHO

## Funding for neglected diseases

Global funding for research and product development for neglected diseases reached a record high of $4.0 billion in 2018 according to the 2019 G-FINDER survey, which reports on global investment in research and development for neglected diseases, including human immunodeficiency virus (HIV), tuberculosis and malaria.

As in previous years, HIV, tuberculosis and malaria collectively received more than two thirds ($2.8 billion, 69%) of all global funding for neglected disease research and development.

http://go.aws/2ugoFtw

**Looking ahead**

2 – 6 March. United Nations Commission on Narcotic Drugs, Vienna, Austria.

4 – 5 March. The WHO Symposium on the Future of Digital Health Systems in the European Region, Copenhagen, Denmark.

9 –11 March. Global Alcohol Policy Conference, Dublin, Ireland.

